# Supplementary material for: Partial cross mapping eliminates indirect causal influences
Source: Nat Commun. 2020 May 26;11:2632. doi: 10.1038/s41467-020-16238-0 (PMC7251131; doi:10.1038/s41467-020-16238-0)
Supplement: Supplementary file 1 — Supplementary Information [file 41467_2020_16238_MOESM1_ESM.pdf]

Supplementary Information for

Partial cross mapping eliminates indirect causal influences

Leng et al.

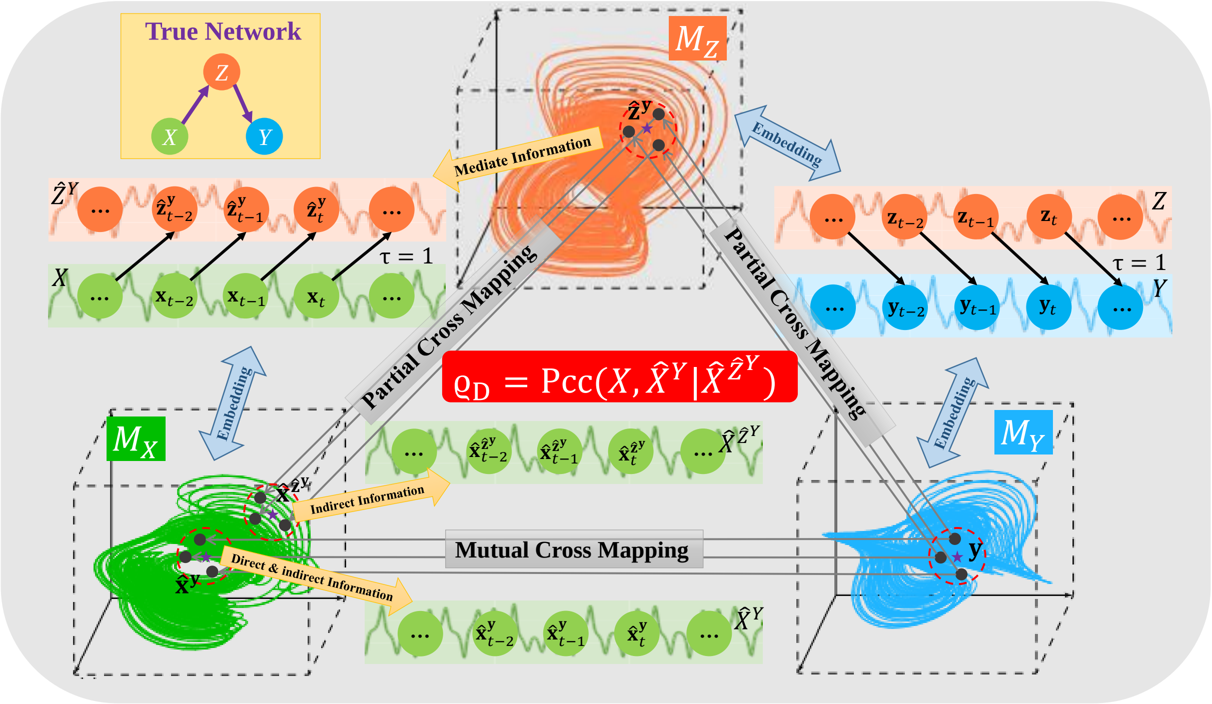

Supplementary Figure 1. A precise illustration for the PCM framework of the three variables interacting in a unidirectional causal chain. Here, the time delay of causal influence is set illustratively as 1. The indirect information is denoted by a successive estimating from M_Y to M_Z then to M_X (the slashed grey arrows labeled with Partial Cross Mapping) and the mapping from M_Y to M_X (the horizontal grey arrows labeled with Mutual Cross Mapping) contains both direct and indirect information. The partial correlation thus eliminates the indirect part. Here in each mapping procedure, an optimal time delay is searched to maximize the information transfer.

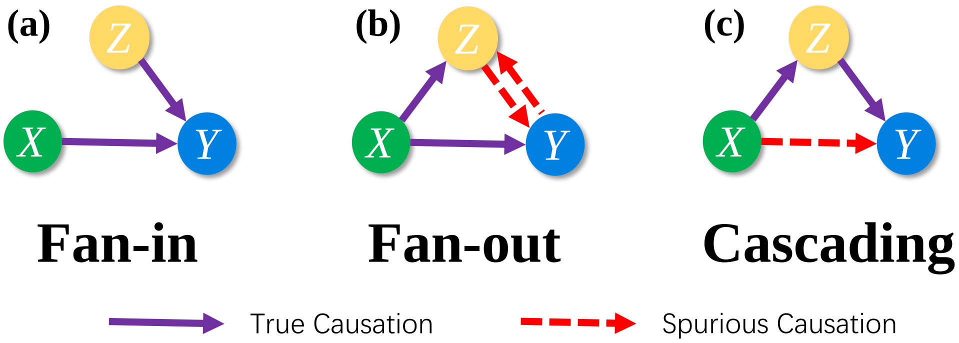

Supplementary Figure 2. Basic network motifs with causation detection. Large-scale networks possess a small set of recurring patterns, i.e., network motifs, which are the basic building blocks of any complex network. The red dashed arrows show spurious causation that easily to be wrongly detected by the existing methods.

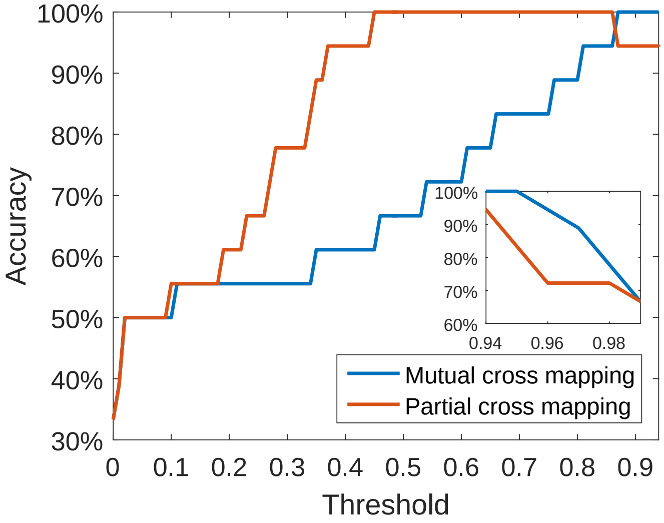

Supplementary Figure 3. Detection accuracy versus threshold T for the PCM and the MCM methods for the benchmark logistic model of three species in the main text. Shown are the accuracy with 18 indices (ϱ_{D} or ϱ_{C}) for all possible links in the three network structures listed in Figs. 1a-c of the main text. Notice that when $T = 0.5$, the MCM method not only misidentifies the indirect causal links, but also reaches wrong results in other links. When $0.95 > T > 0.85$, MCM could achieve 100% accuracy, however, it is not reasonable to choose such a high threshold value larger than 0.85 in practice. Notice that in this benchmark system, the index values (ϱ_{C} or ϱ_{D}) for a true positive direct causal link are of a rather high level, therefore the detection accuracy only decreases when the threshold is approaching 1 (see the partial enlarged view).

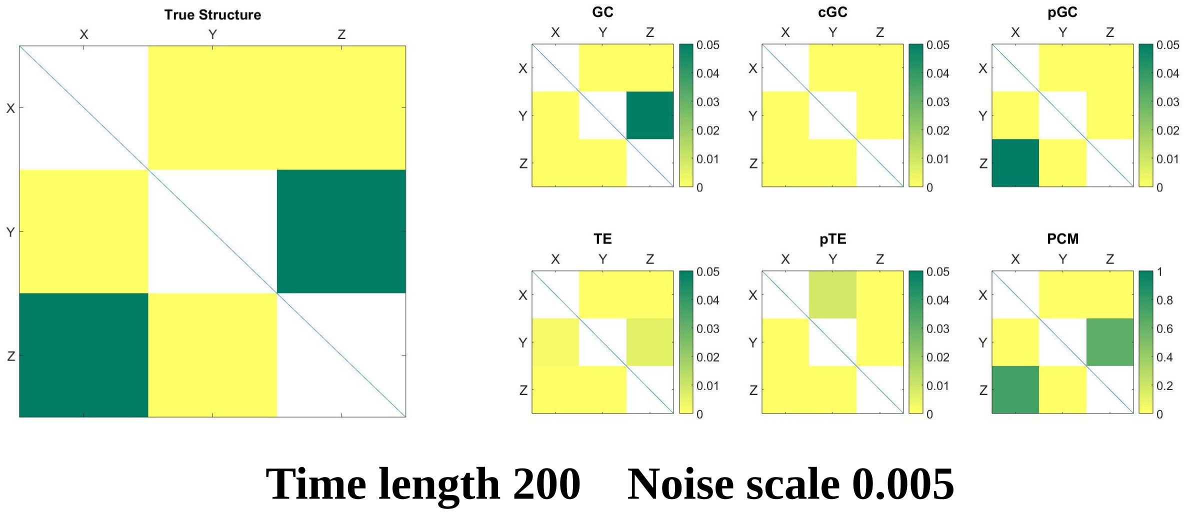

Supplementary Figure 4. Comparison results of the causation detection methods using a model of three species interacting in a chain mode. The true structure is shown by the left panel. The representative methods, including the GC, the cGC, the pGC, the TE, and the pTE, cannot identify the correct structures, while our method, the PCM, is able to identify the true causations correctly and completely. Here the denoted time lengths and the noise scales are relative to the real scale of the system.

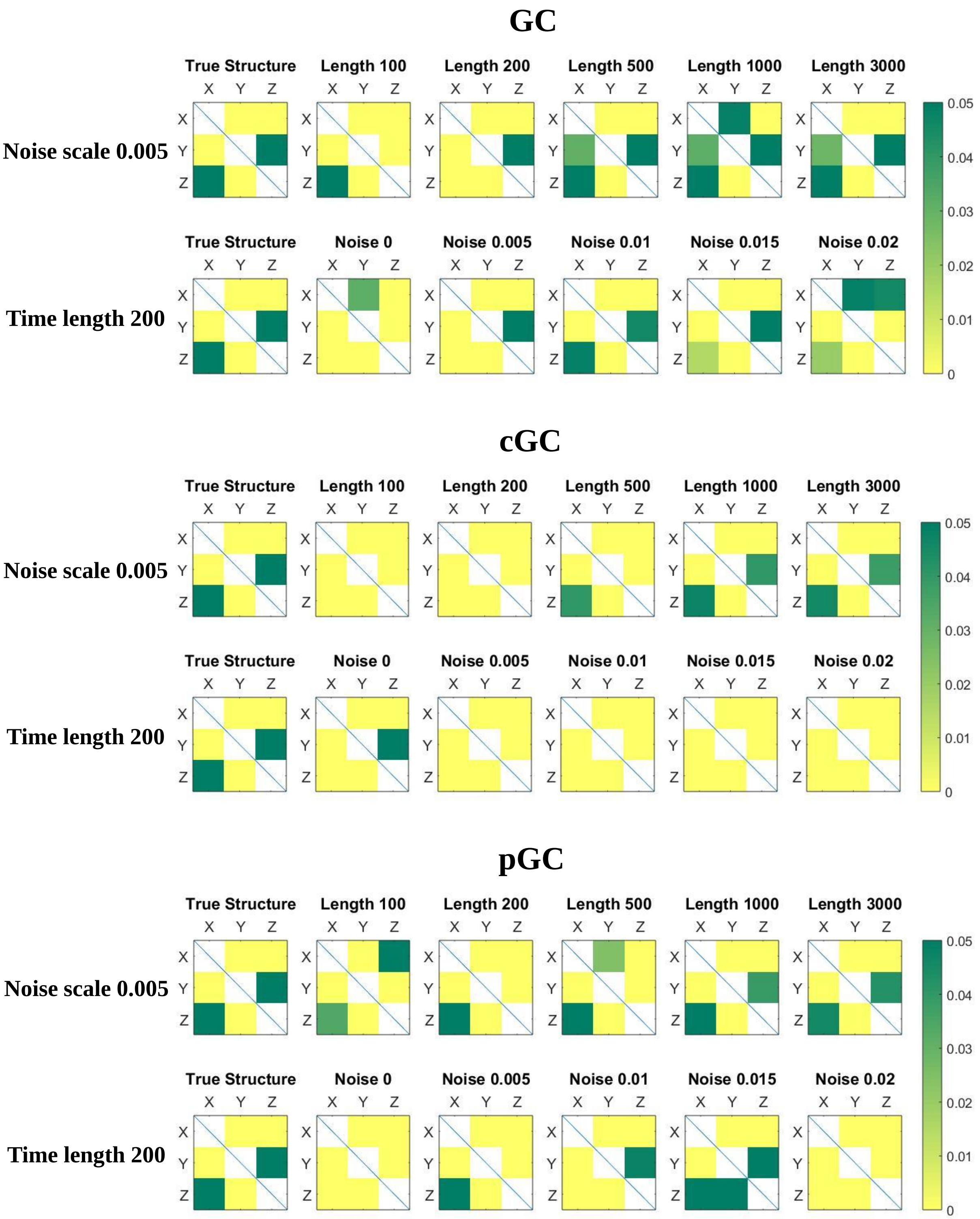

Supplementary Figure 5. Comparison results for causations detection by using the GC, the cGC, and the pGC. Here the denoted time lengths and the noise scales are relative to the real scale of the system.

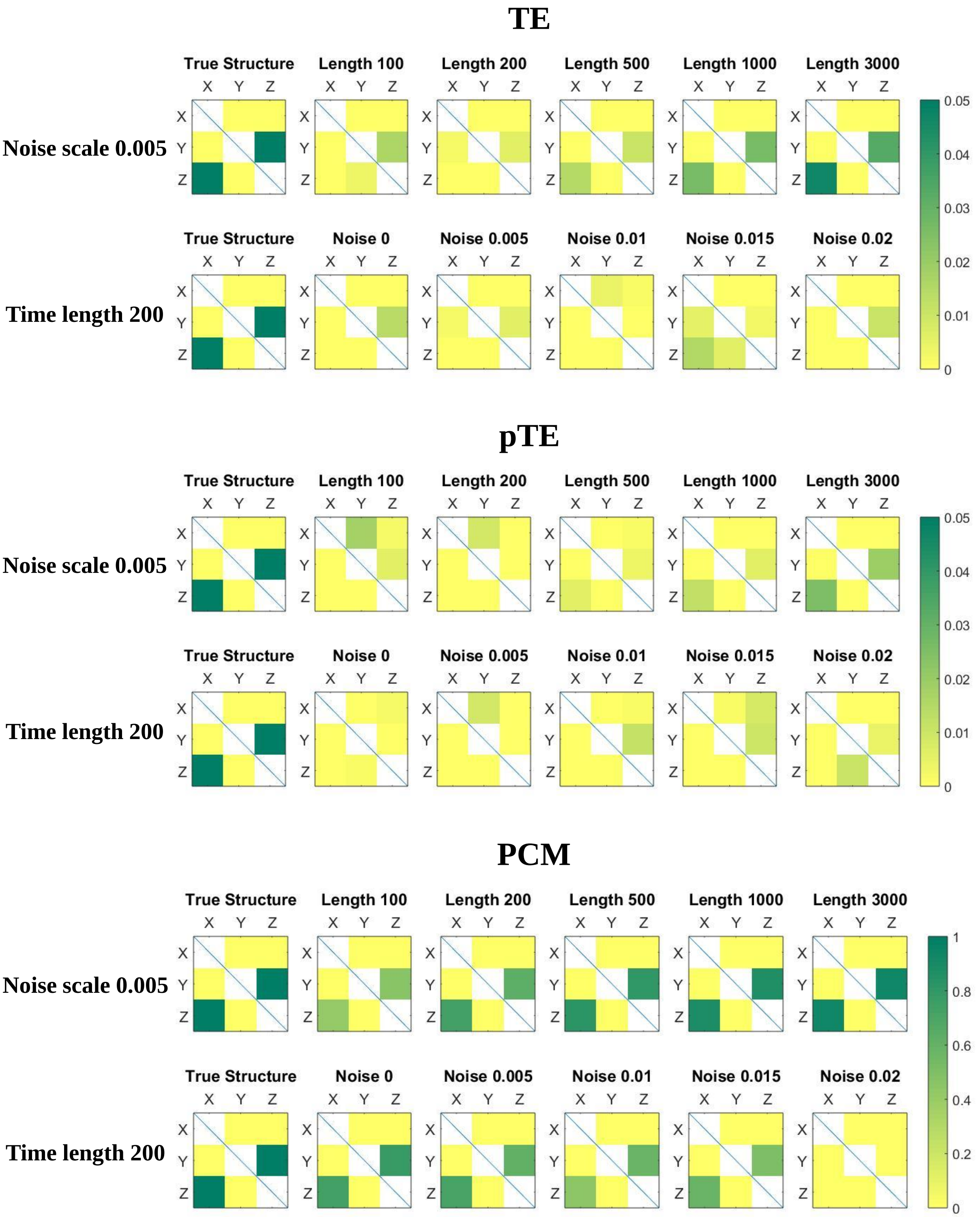

Supplementary Figure 6. Comparison results for causations detection by using the TE, the pTE, and the PCM. The PCM method outperforms all the other methods here and the methods in Supplementary Figure 5 as well. Here the denoted time lengths and the noise scales are relative to the real scale of the system.

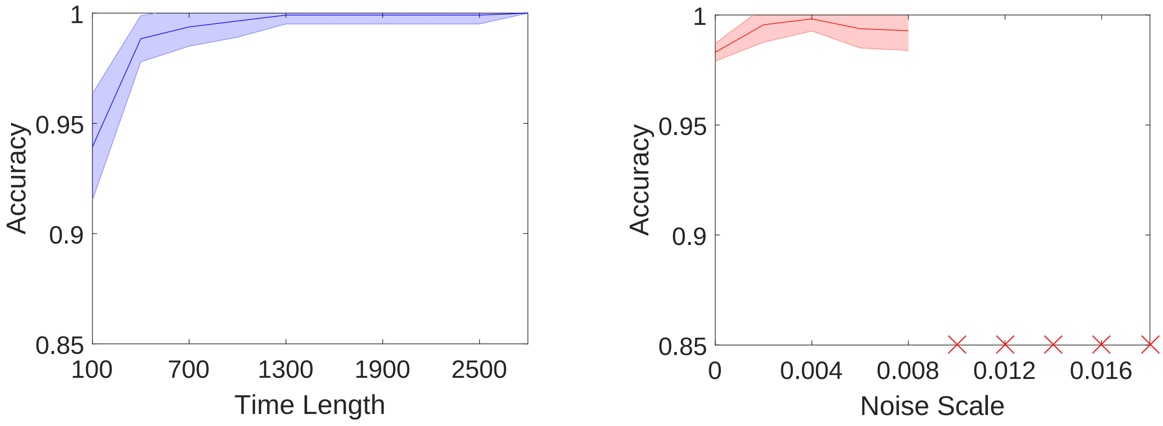

(a) Fixed noise scale 0.002.

(b) Fixed time length 1000.

Supplementary Figure 7. Robustness tests of PCM against time series of different time lengths and noise scales with the networked system consisting of eight species in Supplementary Note 4. The shaded area represents the standard deviation of the PCM results of 100 simulations. The system becomes divergent when the noise scale exceeds 0.01 (shown by "×" on the horizontal axis). Here the time lengths denote the system's absolute time unit and the noise scales are the ratio of the noise strength to the system's amplitude. The threshold value is selected to be 0.5 in this analysis.

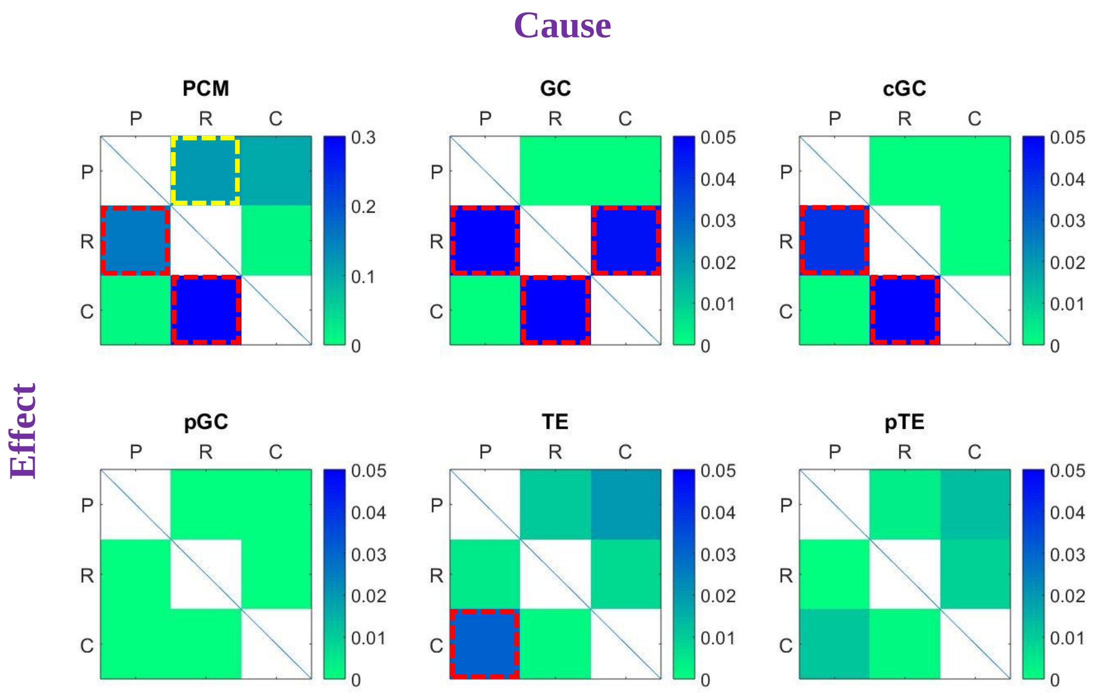

(a) Food chain network of three plankton species.

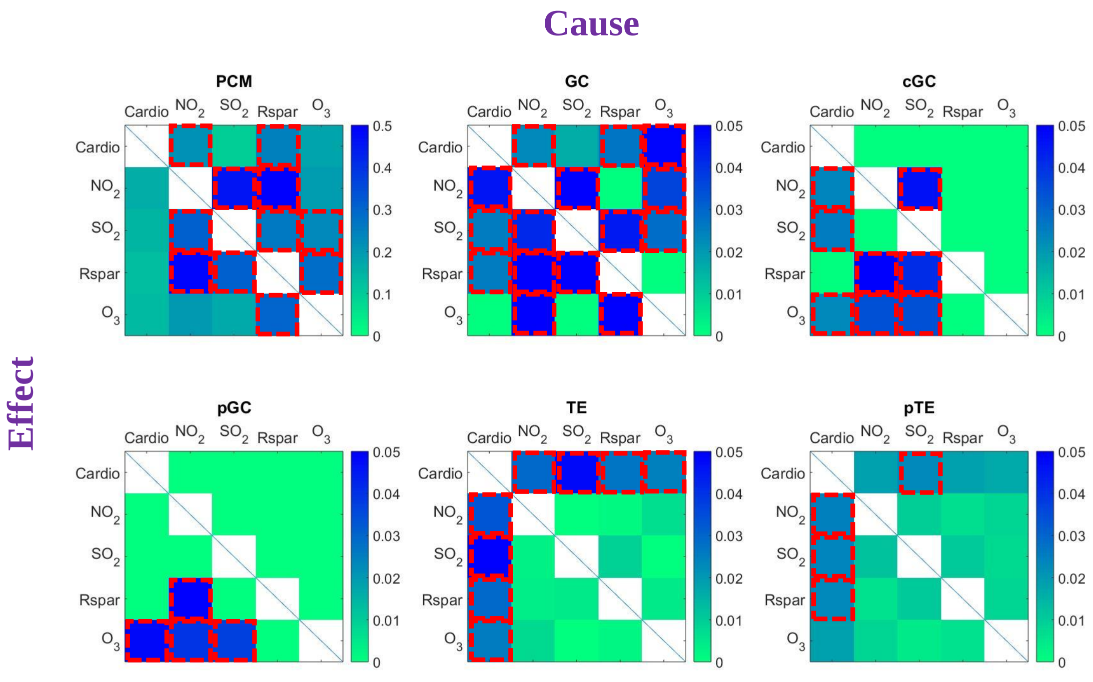

(b) Air pollutants and cardiovascular diseases.

Supplementary Figure 8. Comparison results of causations detection methods with the representative real-world examples. Here, the regions highlighted by the dash boxes correspond to the detected causal links by the respective methods, and the names, *Cyclopoids*, *Rotifers*, and *Pico cyanobacteria*, are abbreviated as C, R, and P, respectively.

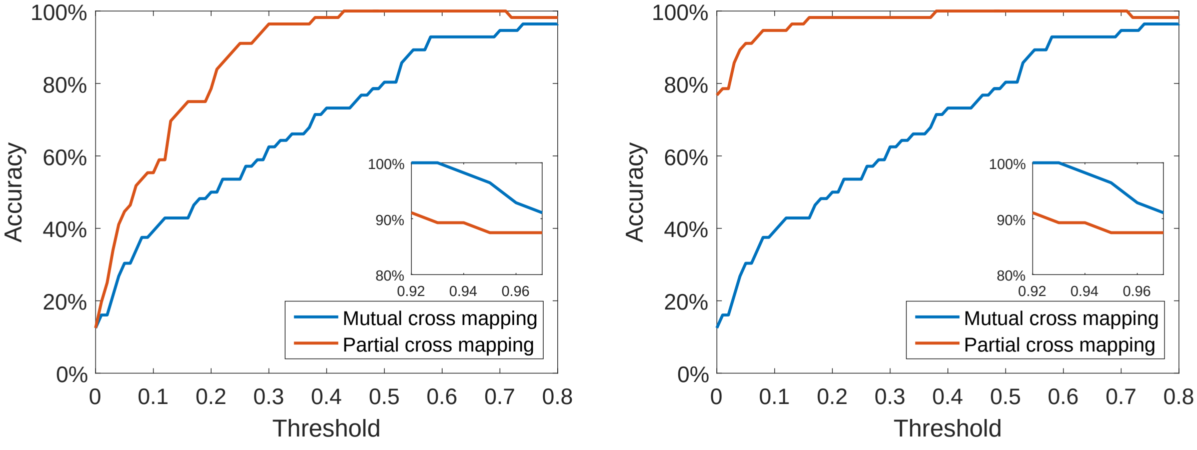

(a) (b)

Supplementary Figure 9. Detection accuracy versus threshold T when the PCM and the MCM methods are in the logistic model Supplementary Equation (4) of eight interacting species. (a) No use of the accessory measure γ. (b) The accessory measure $\gamma \geq 70\%$ is used, which improves the detection accuracy ($\geq 80\%$) for the PCM method for small values of T. The partial enlarged views show that the accuracy decreases when the threshold is too large.

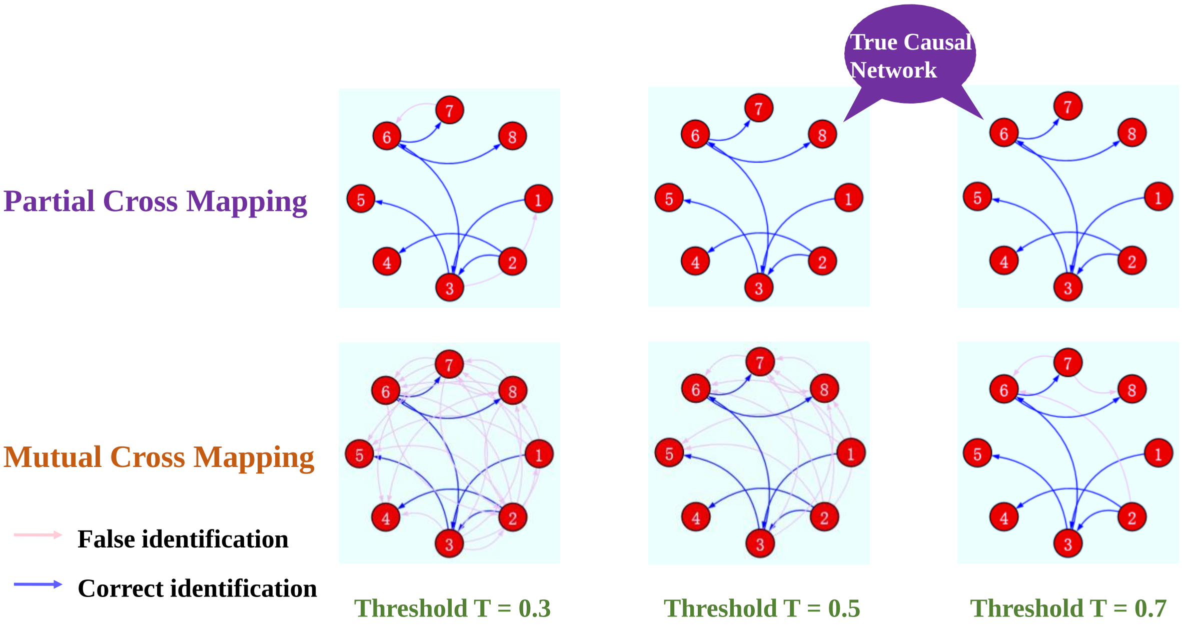

Supplementary Figure 10. Using the PCM and the MCM methods to reconstruct the causal networks, respectively, with different values of T for model Supplementary Equation (4). The blue and pink arrows indicate the correctly and falsely detected direct causal links, respectively.

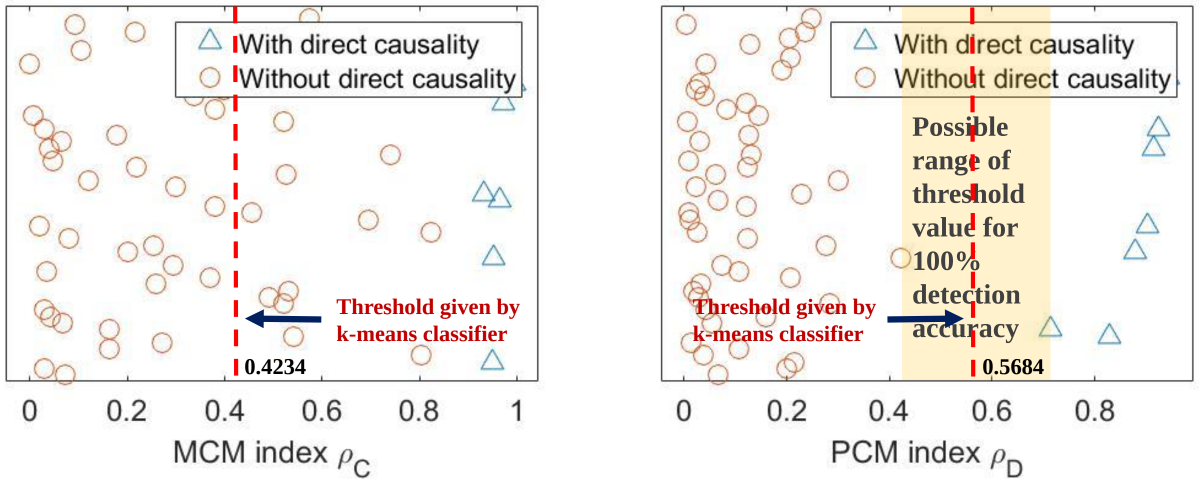

(a) MCM Results **(b) PCM Results**

Supplementary Figure 11. Threshold value selected by k-means classifier for the eight species model. (a) The threshold value induced from k-means classifier is ineffective which produces a lot of false positives for MCM results. (b) For PCM results, the two groups with and without direct causality are distinguished from each other but are concentrated within the interior. This fact facilitates the practical selection of the threshold value and improves the robustness of the PCM method.

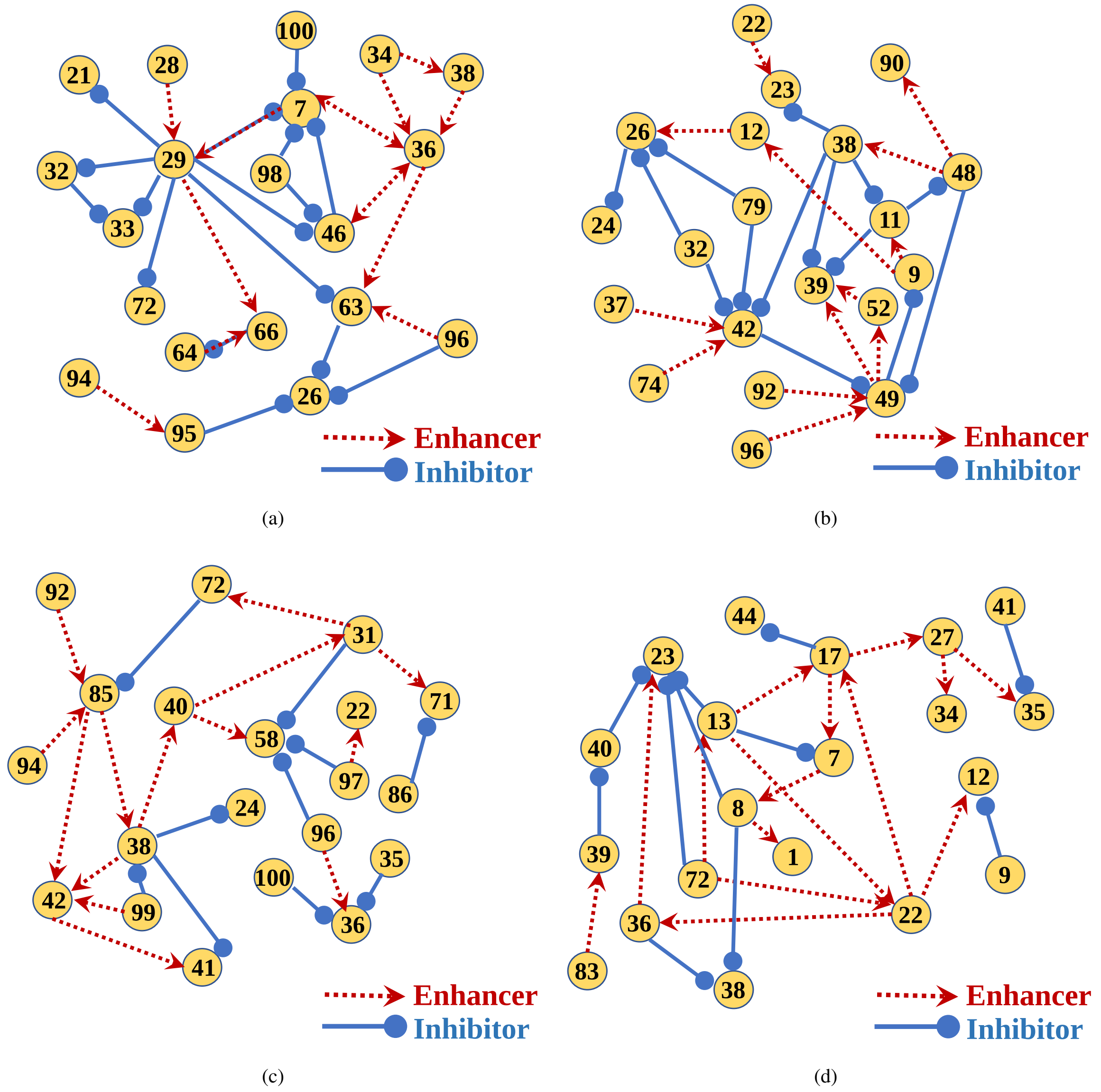

Supplementary Figure 12. Four gene regulatory networks. The four networks together with the one described in the main text [Fig. 4a], with which the PCM and the MCM methods are tested, respectively, in Supplementary Figure 13.

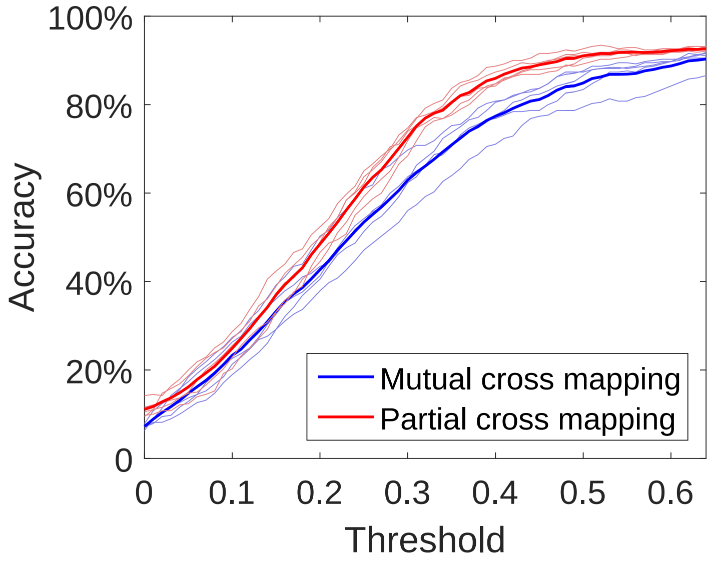

Supplementary Figure 13. Detection accuracy versus the threshold T by using the PCM and the MCM methods for the five gene regulatory networks. The red (blue, resp.) thick curve corresponds to an average detection accuracy of the red (blue, resp.) thin curves, for the five gene regulatory networks by using the PCM (MCM, resp.) method.

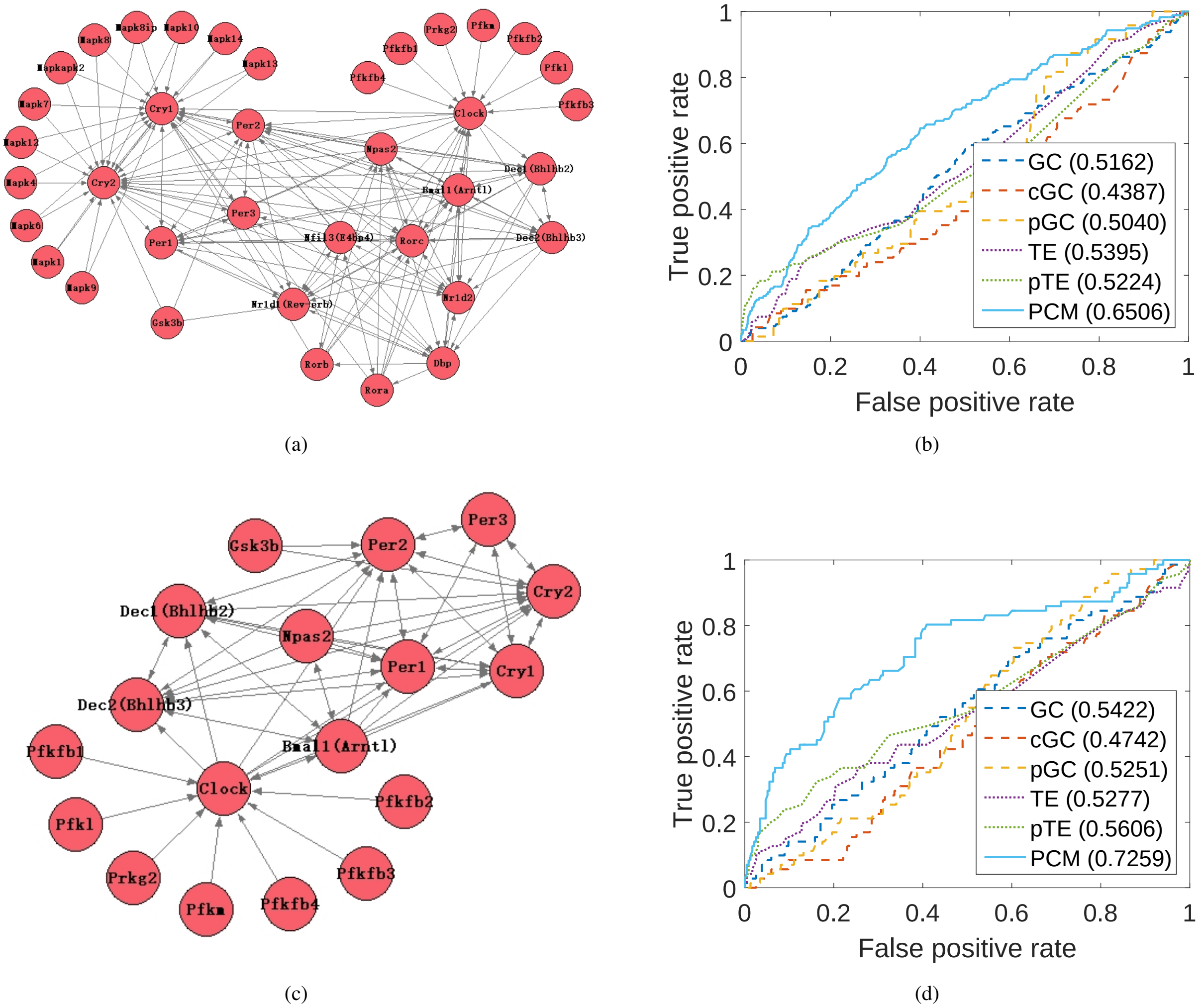

Supplementary Figure 14. Comparison results on causations detection in the real gene regulatory networks containing key genes of circadian rhythms by using different methods. Here, two sub-functional regulatory gene networks with 37 (a) and 18 (c) genes are analyzed, respectively. The PCM method outperforms the other methods in the analyses of the both networks [(b) and (d)]. The phase space reconstruction parameters are taken as $E = 2$ and $\tau = 1$, and the networks are plotted by the software Pajek (http://mrvar.fdv.uni-lj.si/pajek/).

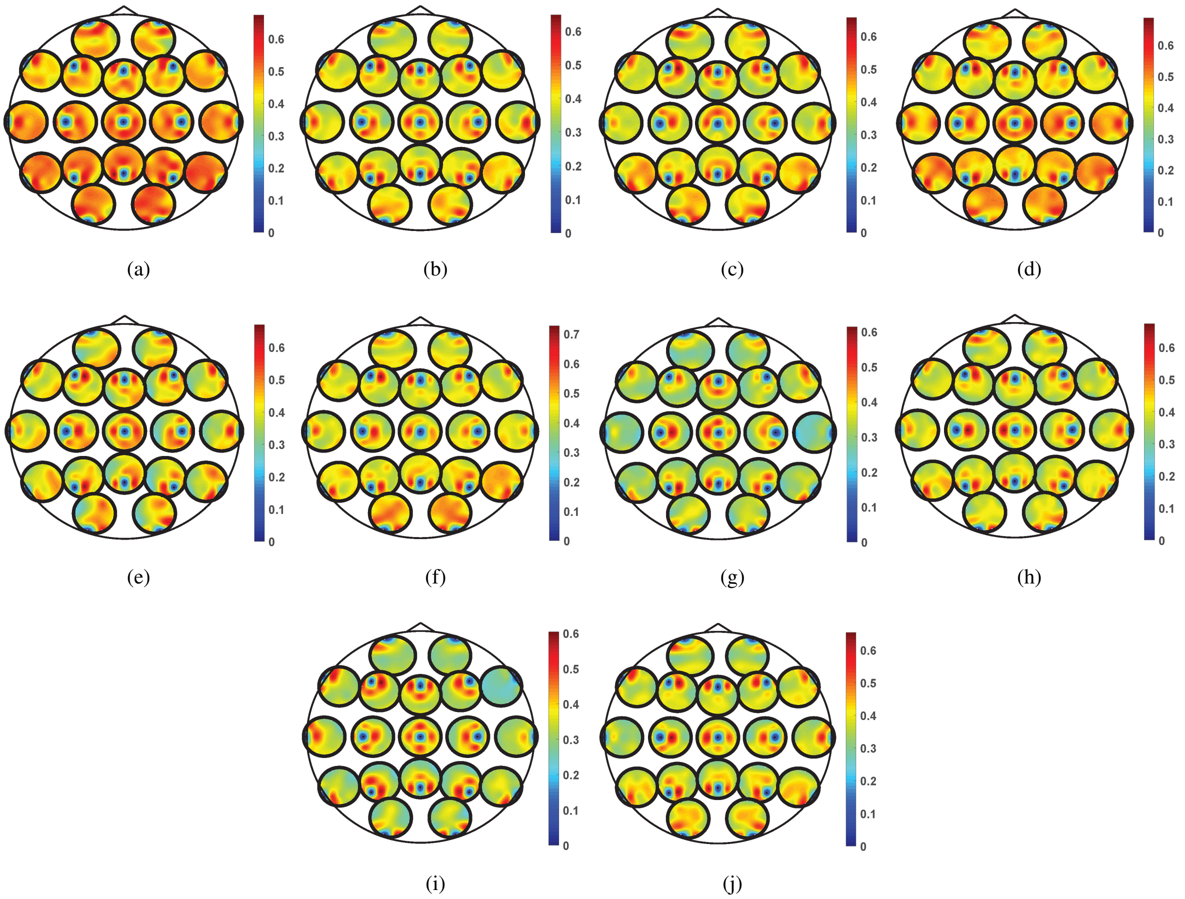

Supplementary Figure 15. The results by using the PCM method on the EEG recordings data for the 10 subjects, respectively. Here, each figure corresponds to an average over 10 randomly selected blocks during which the recording is a continuous measurement that is cleaned of apparent artefacts. In particular, the small circles are positioned at the approximate location of the corresponding electrode placed on the skull. The causation measures from one electrode channel to all the others are displayed at the same relative position within the small circle. The plotting scheme is adapted from [1]. The phase space reconstruction parameters are taken as $E = 6$ and $\tau = 6$.

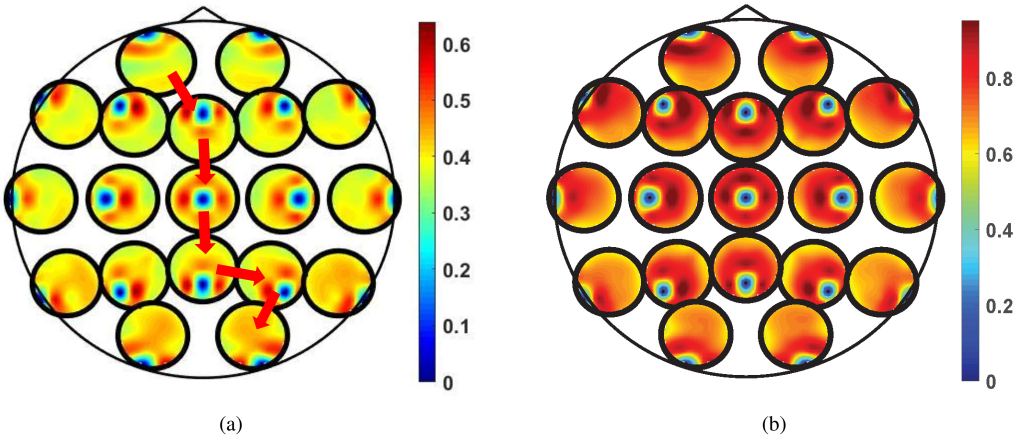

Supplementary Figure 16. Using the PCM method reveals direct electrical information pathways in the human brain. Here, (a) the PCM and (b) the MCM measures are shown between all the possible pairs of 19 channels of the EEG recordings, averaged over 10 subjects and 10 randomly-selected blocks. The detection result using the PCM method is able to reveal the information pathways [e.g., a sample pathway is highlighted by the red arrows in (a)], while the MCM method produces very dense network in every small circle in (b), which indicates the information transmitting in almost every direction but no pathway of specific function.

Supplementary Table 1. Using the PCM method to detect causations for all the possible types of interacting structures of three species. Here, the detecting results are shown, respectively, in the colored matrices, where the direction of each causal interaction is from the variable in the column of the matrix to the different variable in the row. In generating the data of time series, the initial values are taken randomly from the interval $[0, 1]$. The parameters of the phase space reconstruction are taken as: $E = 4$ and $\tau = 1$.

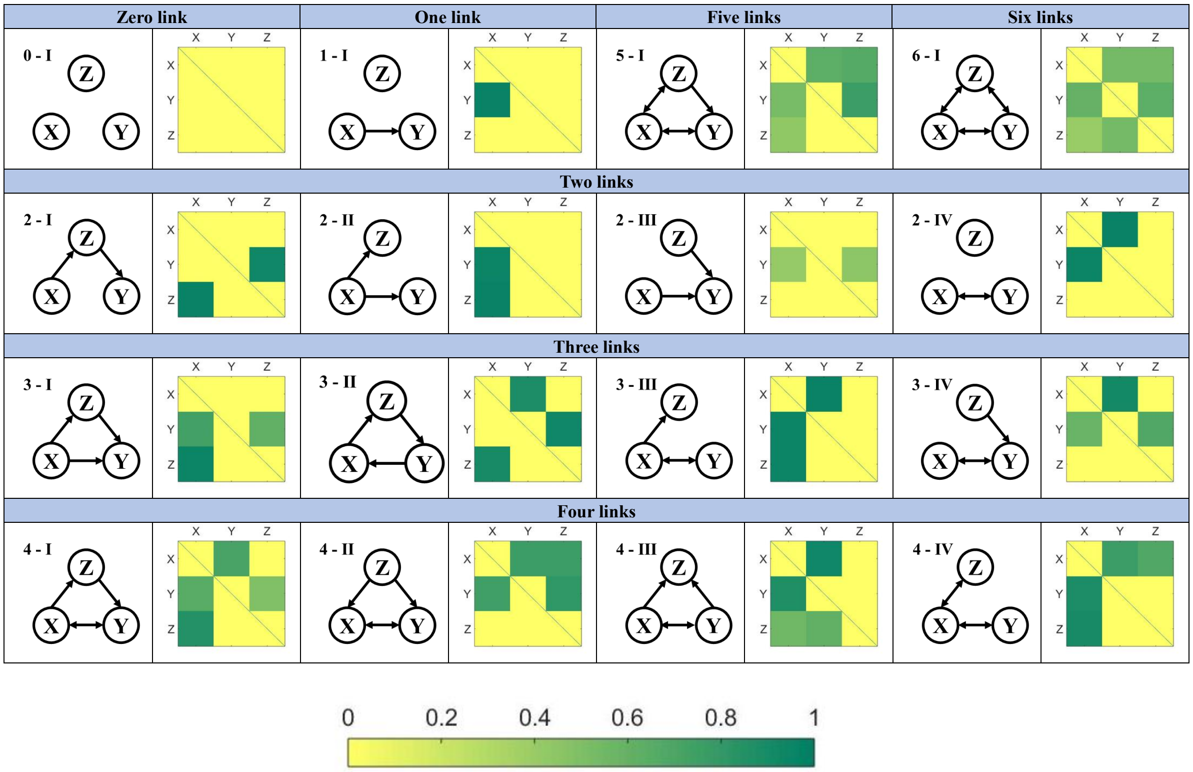

Supplementary Table 2. Measures of all possible causal directions and the corresponding p-values. The system is the benchmark model of three interacting species in the three modes shown in Figs. 1a-c of the main text. The values in bold are the detections for $T \geq 0.5$ ($p \simeq 0$).

Directions	$X \to Y$	$Y \to X$	$X \to Z$	$Z \to X$	$Y \to Z$	$Z \to Y$
MCM in Fig. 1a	$\mathbf{0.9847}(\checkmark)$	0.3493	0.0140	0.0134	6.0100e-04	0.1035
PCM in Fig. 1a	$\mathbf{0.9817}(\checkmark)$	0.3322	0.0137	0.0175	0.0032	0.0961
p-values for PCM	$\mathbf{0}(\checkmark)$	6.9751e-26	0.6727	0.5910	0.9211	0.0031
MCM in Fig. 1b	$\mathbf{0.8681}(\times)$	0.4501	$\mathbf{0.9846}(\checkmark)$	$\mathbf{0.6565}(\times)$	$\mathbf{0.5307}(\times)$	$\mathbf{0.9746}(\checkmark)$
PCM in Fig. 1b	0.1871	0.2262	$\mathbf{0.9573}(\checkmark)$	0.3479	0.3662	$\mathbf{0.9499}(\checkmark)$
p-values for PCM	6.3838e-09	1.7688e-12	$\mathbf{0}(\checkmark)$	2.1943e-28	1.7027e-31	$\mathbf{0}(\checkmark)$
MCM in Fig. 1c	$\mathbf{0.8052}(\times)$	$\mathbf{0.9674}(\checkmark)$	$\mathbf{0.9718}(\checkmark)$	$\mathbf{0.6005}(\times)$	$\mathbf{0.7576}(\times)$	$\mathbf{0.9593}(\checkmark)$
PCM in Fig. 1c	0.4467	$\mathbf{0.9590}(\checkmark)$	$\mathbf{0.8609}(\checkmark)$	0.2725	0.2685	$\mathbf{0.9480}(\checkmark)$
p-values for PCM	9.7651e-48	$\mathbf{0}(\checkmark)$	$\mathbf{3.8035e-280}(\checkmark)$	1.2785e-17	3.9153e-17	$\mathbf{0}(\checkmark)$

Supplementary Table 3. The scope of the GC-based, TE-based and PCM causation detection methods. The abbreviations could be find in Supplementary Note 3.

	Separable system	Non-separable system
Pairwise	GC, cGC, pGC, TE, pTE	MCM, PCM
Network	cGC, pGC, pTE	PCM

Supplementary Table 4. For the PCM method, the p-values associated with the seven detected direct causal links for the logistic model of the eight interacting species. Here, all the p-values for the true links are approximately zero, showing high statistical significance and accurate detection results as the PCM method is used.

Directions	$1 \to 3$	$2 \to 3$	$2 \to 4$	$3 \to 5$	$3 \to 6$	$6 \to 7$	$6 \to 8$
p-values	$\mathbf{0}$	$\mathbf{9.8212e-148}$	$\mathbf{0}$	$\mathbf{0}$	$\mathbf{1.7441e-306}$	$\mathbf{0}$	$\mathbf{8.2680e-240}$

Supplementary Table 5. For the food chain network of three plankton species, the p-values associated with all possible causal directions. The names of *Cyclopoids*, *Rotifers*, and *Pico cyanobacteria* are abbreviated as C, R, and P, respectively. The values in bold are the detections for $T \geq 10^{-1}$ ($p < 0.0016$).

Directions	C $\to$ R	R $\to$ C	C $\to$ P	P $\to$ C	R $\to$ P	P $\to$ R
p-values	0.7495	$\mathbf{1.1302e-17}$	0.0054	0.8259	$\mathbf{0.0011}$	$\mathbf{1.2119e-05}$

Supplementary Table 6. PCM generated p-values for all possible causal directions between the air pollutants and the admission numbers of cardiovascular diseases in major hospitals in Hong Kong. The values in bold are the detections for $p < 10^{-9}$.

Directions	Cardio $\to NO_2$	$NO_2 \to$ Cardio	Cardio $\to SO_2$	$SO_2 \to$ Cardio
p-values	1.1268e-06	$\mathbf{4.0209e-11}$	2.0593e-05	0.0038
Directions	Cardio $\to$ Rspar	Rspar $\to$ Cardio	Cardio $\to O_3$	$O_3 \to$ Cardio
p-values	8.7456e-05	$\mathbf{3.3594e-14}$	5.0206e-05	6.7548e-08
Directions	$NO_2 \to SO_2$	$SO_2 \to NO_2$	$NO_2 \to$ Rspar	Rspar $\to NO_2$
p-values	$\mathbf{8.6071e-21}$	$\mathbf{6.8415e-47}$	$\mathbf{6.5167e-55}$	$\mathbf{4.0150e-63}$
Directions	$NO_2 \to O_3$	$O_3 \to NO_2$	$SO_2 \to$ Rspar	Rspar $\to SO_2$
p-values	2.8376e-09	6.9308e-09	$\mathbf{3.3639e-21}$	$\mathbf{8.1370e-16}$
Directions	$SO_2 \to O_3$	$O_3 \to SO_2$	Rspar $\to O_3$	$O_3 \to$ Rspar
p-values	2.3718e-07	$\mathbf{1.7075e-12}$	$\mathbf{2.6498e-20}$	$\mathbf{2.0512e-18}$

Supplementary Note 1. Detecting causations in all types of structures for three interacting species

In addition to the three interacting structures for three species in the main text, our proposed PCM method is effectively applicable to detecting causations for all the other types of interacting structures as listed in Supplementary Table 1. Particularly, as is well-known, the structures, such as the fan-out spurious causation due to the common source [Supplementary Table 1 #2-II and Supplementary Figure 2(b)] and the indirect spurious causation due to the cascading [Supplementary Table 1 #2-I and Supplementary Figure 2(c)], always result in false detections when the existing methods are used. However, our PCM can achieve in distinguishing these spurious links. Additionally, these two spurious links, with the fan-in mode [Supplementary Table 1 #2-III and Supplementary Figure 2(a)], become the elementary structures for building blocks in any complex networks [2, 3]. Since the PCM method can achieve accurate detections of causations in these elementary modes, using the PCM method to reconstruct causal networks of large scale becomes doable and reliable.

In our systematic analyses, the species dynamics of X, Y, and Z are taken as the logistic maps with unchanged parameters for all the possible interacting structures. More precisely, the model of the interacting species is:

$$x_t = x_{t-1}(\alpha_x - \alpha_x x_{t-1} - \beta_{xy} y_{t-1} - \beta_{xz} z_{t-1}) + \epsilon_{x,t-1}, \quad (1)$$

$$y_t = y_{t-1}(\alpha_y - \alpha_y y_{t-1} - \beta_{yx} x_{t-1} - \beta_{yz} z_{t-1}) + \epsilon_{y,t-1}, \quad (2)$$

$$z_t = z_{t-1}(\alpha_z - \alpha_z z_{t-1} - \beta_{zx} x_{t-1} - \beta_{zy} y_{t-1}) + \epsilon_{z,t-1} \quad (3)$$

with the parameters that are set, respectively, as $\alpha_x = 3.7$, $\alpha_y = 3.78$, and $\alpha_z = 3.72$. The coupling strength are taken as 0.35 or 0 to form the corresponding structure. The last term $\epsilon_{i,t}$ $(i \in \{x, y, z\})$ in the above equations are the white noise with the strength 0.005 (see Supplementary Note 3 for detailed information on the scale of the noise). We consider 100 trails with the length of 1000 points, which are randomly selected from the generated time series with the length of 5000 points. Here, the sampling rate is 1 Hz, so the number of time points matches exactly the time unit of the system. The average of the PCM index for each possible causal direction over these 100 trails is shown in Supplementary Table 1.

Supplementary Note 2. Additional information for benchmark systems in the main text

As described in the main text, the value of the threshold T is set to be 0.5. The PCM method reconstructs correctly the causal networks of different structure for the benchmark system. How does the choice of the threshold T affect the detection accuracy for PCM or the previous MCM method? For the benchmark systems in main text [Fig. 3], we show in Supplementary Figure 3, for both methods, the detection accuracy varies as the threshold value is increased. Here detection accuracy denotes total correct classification rate (both negative and positive) of direct causal links. However, the range of T values in which the detection accuracy approaches 100% is much broader for PCM than for MCM, demonstrating the robustness and broad applicability of the PCM method to real-world systems when *a priori* knowledge for prescribing the value of T is lacking.

For data integrity and statistical significance, we list in Supplementary Table 2 all the computational results for all the possible causal directions for the three modes in Figs. 1a-1c [or Fig. 3a] in the main text. Also listed in this table are the results on the corresponding p-values for the PCM measures, where the p-values are obtained by testing the hypothesis of no partial correlation against the alternative that there is non-zero partial correlation. The final results are all verified by multi-testing corrections via the Bonferroni adjustment ($p < \alpha/n$), where n is the number of the edges in the network [4, 5].

Supplementary Note 3. Systematic comparison of the PCM method with the representative methods

Presently, there are three major paradigms for identifying and quantifying causal interactions: the celebrated Granger causality, the entropy based methods, and the mutual cross mapping. The Granger causality and the entropy based methods are likely to work ineffectively in the situation where the relevant dynamical variables are

non-separable. As a matter of fact, in the benchmark and real-world nonlinear dynamical systems, which are also the systems we are mainly investigating in this work, the non-separable variables are dominantly present. The mutual cross mapping based methods allow for detecting causations between the non-separable variables but cannot distinguish direct causation from indirect ones (as described and compared in the main text). Here, we perform systematic comparison studies on the performances of the representative methods in the literature and our PCM method using the benchmark systems and the real-world examples. The scope of the methods investigated here are summarized in Supplementary Table 3.

3.1. Benchmark systems with different time lengths and different noise scales

We still use the coupled logistic maps Supplementary Equations (1)-(3) as a benchmark system to perform the comparison study. We consider the causal chain mode $X \to Z \to Y$ where $\beta_{yz} = 0.1$, $\beta_{zx} = 0.1$, and the other coupling parameters are set as zero. Different lengths of time series (i.e., $100, 200, 500, 1000, 3000$) and different noise levels (i.e., $0, 0.005, 0.01, 0.015, 0.02$) are, respectively, taken into account. Here, the sampling rate is 1 Hz, so the number of time points matches exactly the time unit of the system. We will use the term "time length" to denote the absolute length of the time duration for a time series. Additionally, we generate the white noises with respect to each time unit and use the term "noise scale" as the ratio between the noise strength and the amplitude of the system dynamics (e.g., the amplitude is 1 for the logistic map, so the noise strength is exactly the same as the noise scale here). We compute the causation index for all the six possible causation directions by using the following methods, respectively: the Granger causality (GC), the conditional Granger causality (cGC), the partial Granger causality (pGC), the transfer entropy (TE), the partial transfer entropy (pTE), and our method, the PCM. The realization details for all the methods, the significance test methods, and the other numerical configurations are summarized in Supplementary Note 5.

We first compare the six methods using the time series with the time length of 200 and with the noise scale 0.005 as well. As shown in Supplementary Figure 4, only the proposed PCM method is able to identify the correct causation structure of the unidirectional chain, while the other methods are all failed in finding the correct structure.

Next, we test the above-mentioned methods with different time lengths and different noise scales. We then illustrate the range of application for each method in details. For the original GC, the detection results are quite unstable [Supplementary Figure 5], which is due to the non-separable variables are present in our nonlinear system. As a matter of fact, the Granger causality method and its extensions (the cGC and the pGC), which are all based on regression, are theoretically unsuitable for causation detection for nonlinear systems with non-separable variables. Although the conditional and the partial Granger causality tests perform well only when the time length is sufficiently long, the detection results are not based on the solid ground. This is because the regression actually produces a tremendously large deviation from the true dynamics in the first step of the GC detection where a presumed regression model is required to fit with the observed time series. Such a fitting deviation definitely renders the results obtained by the GC method unreliable. As for the data produced by a presumed model with strong stochasticity, the GC as well as its extensions is still regarded as the most useful technique in causation detections. However, as for the data produced by any unknown dynamical model with weak and moderate stochasticity, our PCM method is more reliable and effective due to its solid ground of dynamical systems theory.

The TE method was developed to deal with both linear and nonlinear systems. For the special case of the Gaussian variables, the GC and the TE are in fact equivalent to each other [6]. However, the TE and the pTE methods require reconstruction of the probability distributions of the pertinent variables from the observed data. The performance of causation detections by using these two method thus significantly depend on the time length of the available time series, as shown in Supplementary Figure 6. Moreover, it is emphasized that, theoretically, neither the TE nor the pTE allow for any non-separate variables in causation detections.

However, for our proposed PCM method, we could easily find that PCM indeed outperforms the other representative frameworks of causation detections. PCM is not restricted by the separability condition and effective even when the time length is not sufficiently long. However, the PCM is sensitive to large noise, and will produce false negative detections which is inevitable.

To further explore the robustness of the PCM framework against time series lengths and the noise scales, we performed an additional numerical analysis using the eight species system introduced in Supplementary Note 4. As expected, Supplementary Figure 7 shows that the detection accuracy increases with the time length of the time series used in simulations but always remains at a high level, confirming the effectiveness of our PCM framework in dealing with an extremely small amount of data. In addition, increasing the noise scale only slightly lowers the

detection accuracy. Note that the system becomes divergent as the noise scale is larger than 0.01 [shown by "×" on the horizontal axis in Supplementary Figure 7(b)]. These also demonstrate that our PCM framework is useful when the noisy perturbation is introduced in a manner of small or moderate intensity.

3.2. Comparison on real-world examples

We will compare our PCM method with other causal assessment methods also on the real-world examples. Here we perform the comparison with the three plankton species food chain network and the example of air pollution with cardiovascular diseases.

As shown in Supplementary Figure 8, the GC and cGC could successfully identify the chain structure among the three species, but fail to detect the weak causation from *Rotifers* to *Pico cyanobacteria*. However, GC even produces a false link from *Cyclopoids* to *Rotifers*, which shows GC is unsuitable for detecting causality in this system. Moreover, pGC, TE and pTE can hardly detect the structure and even produce false positive. As to the second example, methods except PCM all find causal links from cardiovascular diseases to the air pollutants, which is unreasonable to the true network. And cGC and pGC fail to find any causal relation from air pollutants to cardiovascular diseases, which has been proved in literature [7, 8].

3.3. Comparison of PCM and DBI

First, the method of the dynamical Bayesian inference (DBI) does not require the detailed knowledge of the explicit equations in the models but only uses a delicate selection of a basis set in some function space for data regression [9, 10, 11, 12]. It is applicable for general autonomous and nonautonomous systems. Our PCM framework is a model-free method, only based on the embedding theorem, which is theoretically suitable for dealing with autonomous systems or/and nonautonomous systems with some particular forms as mentioned in the main text. More concretely, the PCM framework works for the switching systems where the switching points can be located and each duration between the consecutive switching points is sufficiently long, while the DBI is applicable for more types of nonautonomous systems, including the dynamical oscillators with time-evolving coupled functions or/and with various types of noise [9]. Second, the DBI method can infer the exact coupling functions and underlying dynamical mechanisms, while our PCM framework focuses much on the detection of causal relations. So, the connections detected by the DBI could be regarded as effective connectivity while the causal relations found by the PCM framework are more like functional connectivity [13]. Additionally, our PCM is able to distinguish direct causations from indirect ones, while the DBI method could be further extended to a conditional version. Both methods have their own particular advantages and could be used in a complementary manner. For example, in highly complex networks, our PCM framework can first detect the basic network structures based on the observed data, significantly simplifying initial regression structure with the function basis set.

Supplementary Note 4. Reconstruction of direct causal networks of eight benchmark species

We consider the system

$$\begin{array}{rcl} x_1(t) &=& x_1(t-1)[3.9 - 3.9x_1(t-1)] + \epsilon_{1,t}, \\ x_2(t) &=& x_2(t-1)[3.5 - 3.5x_2(t-1)] + \epsilon_{2,t}, \\ x_3(t) &=& x_3(t-1)[3.62 - 3.62x_3(t-1) - 0.35x_1(t-1) - 0.35x_2(t-1)] + \epsilon_{3,t}, \\ x_4(t) &=& x_4(t-1)[3.75 - 3.75x_4(t-1) - 0.35x_2(t-1)] + \epsilon_{4,t}, \\ x_5(t) &=& x_5(t-1)[3.65 - 3.65x_5(t-1) - 0.35x_3(t-1)] + \epsilon_{5,t}, \\ x_6(t) &=& x_6(t-1)[3.72 - 3.72x_6(t-1) - 0.35x_3(t-1)] + \epsilon_{6,t}, \\ x_7(t) &=& x_7(t-1)[3.57 - 3.57x_7(t-1) - 0.35x_6(t-1)] + \epsilon_{7,t}, \\ x_8(t) &=& x_8(t-1)[3.68 - 3.68x_8(t-1) - 0.35x_6(t-1)] + \epsilon_{8,t}, \end{array} \tag{4}$$

where $\epsilon_{i,t}$ $(i = 1, \ldots, 8)$ terms are white noise of zero mean and standard deviation 0.005 (see Supplementary Note 3 for detailed information on the scale of the noise). Supplementary Figure 9 shows the detection accuracy versus the threshold T for both the PCM and MCM methods. The PCM method gives much better performance in

reconstructing the direct causal networks. The accessory measure γ introduced in the main text is also shown in Supplementary Figure 9. Taking γ into account can improve the detection accuracy for small values of T.

Supplementary Figure 10 presents the reconstructed direct causal networks for three different values of T: $T = 0.3$, $T = 0.5$, and $T = 0.7$. We see that, even for $T = 0.3$, the PCM method recovers almost all the direct causal links, and for $T = 0.5$ and $T = 0.7$, the reconstruction error is essentially zero. In contrast, the MCM method gives dense networks containing many indirect causal links and false direct links even for T above 0.5. Supplementary Table 4 lists the p-values verified by the multi-testing corrections for the seven direct causal links.

It is worthwhile to mention that using the PCM method with a larger value of T does not always result in the most accurate detection of causations not only for the data that are generated by dynamical models (see Supplementary Figures 3 & 9) but particularly for the real-world data where the internal or external perturbations are unavoidable. The PCM measurement that we design actually quantifies the association between the variables whose interacting structure is even nonlinear. Such a nonlinear interaction, together with computation of normal/partial correlation and various types of perturbations, are likely to reduce to some extent the absolute value of our measurement, which makes the selection of a larger value of T impractical for causations detection in real applications. Therefore, the results in Supplementary Figure 10 manifest that the PCM method does not require the use of a larger value of T, more applicable to applications. The effective selection of threshold value is also much more realizable and robust for our PCM method than for MCM method, see Supplementary Note 5 and Supplementary Figure 11.

Supplementary Note 5. Quick tips for parameter selections, significance tests, and numerical configurations

Q1. How to determine computationally the embedding dimensions and time lags in the phase space reconstruction?

A1. A theoretical requirement guaranteeing the topological equivalency between reconstructed manifold and the original attractor is that the embedding dimension is twice lager than the fractal dimension of the attractor. In practice, we use the method of false nearest neighbor (FNN) and delayed mutual information (DMI) to determine the embedding dimensions and time lags respectively. Moreover, method of linear autocorrelation or other advanced techniques can also be applied in determining these parameters [14, 7]. In the EEG example in Supplementary Note 7, the embedding parameters are adapted from [15]. All these parameters are listed in the captions of the corresponding figures.

Q2. How many nearest neighbors are used in the mutual cross mapping procedure?

A2. We use $E + 1$ nearest neighbors (E is the embedding dimension), which is the minimum number of points needed for a bounded simplex in an E-dimensional space.

Q3. How to determine the threshold value T in practice?

A3. The selection of the threshold is indeed to some extent empirical. In this research, we provide robustness tests on the detection accuracy versus different threshold levels, which shows a practically effective threshold value really exists in a rather wide range (refer to Supplementary Figures 3, 9, 10, 13). Moreover, we additionally provide possible methods regarding the selection of threshold in practice. Unsupervised classifiers such as k-means clustering [16] could be applied to determine the threshold value by classifying the detection results into two groups (i.e., with or without direct causation). And the t-test could also be performed to determine the significance level of the difference between pairs of nodes with or without direct causal relation, while a low p-value represents the two groups are distinguished from each other but concentrated within the interior, thus the selection of a threshold is indeed realizable. As an example, we show in Supplementary Figure 11 the threshold values selected by k-means clustering for classification method for the MCM and PCM detection results of the eight species model discussed in Supplementary Note 4, where results for all possible causal directions are denoted. For the MCM results, though the true positive direct causal links have relatively higher index values, it is difficult to clearly distinguish the two groups with or without direct causality, and the threshold value induced from k-means classifier is ineffective which produces a lot of false positives. However, our PCM method successfully eliminates indirect causation with a distinguishable difference between the two groups. The

threshold value automatically induced from k-means classifier achieves 100% detection accuracy. Moreover, the possible range of threshold value for 100% detection accuracy is quite large, which facilitates practical selection of the threshold value and improves the robustness of the PCM method. T-test also reveals a significant difference in PCM detection results between pairs of nodes with or without direct causal relation with $p < 6.2397\mathrm{e}-25$ in all possible directions of the network. All these results provide effective methods in selecting the threshold value robustly in practice.

Q4. How long is the time series used in the numerical experiments?

A4. If not specified in the context of the corresponding experiments, all the simulations utilize 1000 time length series (with the sampling rate 1 Hz, so that the length matches exactly the time unit of the system).

Q5. How to guarantee the robustness of the numerical experiments?

A5. For all the examples of toy models, randomly selected are the 100 trials with a certain time length (usually 1000 time points if not specified) from 5000-length time series (here, the sampling rate is 1 Hz, so that the length matches exactly the time unit of the system). The average is calculated over results on these randomly selected trials. All the results are verified by the multi-testing corrections via the Bonferroni adjustment ($p < \alpha/n$), where n is the number of the edges in a network.

Q6. What are the numerical configurations and software packages used in the comparison studies?

A6. For the GC evaluations, we perform a standard GC test (i.e., F-test) and set the critical value of the significance (i.e., p-value) as 0.05. For consistency, we show the value $(0.05 - p)$ in the figures of relevance to represent the GC test results. We estimate and test the cGC and the pGC by using the MVGC Multivariate GC Toolbox v1.0 [17] and show in the figures of relevance the product of the index value and the significance value (i.e., 1 or 0). For the data from the toy model, we use the criterion, the AIC, to determine the model order with a ceiling value 10 time units when the GC, the cGC, the pGC are taken into account. However, for real-world data set, we determine the model order by AIC with the ceiling orders as, respectively, 5 time units for the DREAM4 GRN example, 60 time units for the air pollution with cardiovascular diseases system, 15 time units for the real gene regulatory network example, and 25 time units for the EEG example (the corresponding time units are stated in the text of each example). We normalize (i.e., detrend and demean) the time series when the GC method and its extensions are used in causations detection. For the TE evaluations, we utilize the package, TIM Matlab 1.2.0 (http://www.cs.tut.fi/ timhome/tim/tim.htm). The evaluation procedure actually begins with the reconstruction of the state space also using the delay-coordinate embedding technique, which is the same as the phase reconstruction step in the PCM framework. The package uses 20 nearest neighbors and the mutual information to determine the value of the model lag (see the user documentation http://www.cs.tut.fi/ timhome/tim/user_documentation.htm for details). The maximal lags are determined as 1, 10, 15, and 60 time units, respectively, for the logistic model, the plankton system, the real gene regulatory networks, and the air pollution with cardiovascular diseases system. Additionally, we use the permutation test to distinguish the causal link whose significance (p-value) is smaller than 0.05. The configurations of the pTE evaluations are in the almost same manner as those of the TE evaluations where only the estimator is changed to the partial version. For the case where the number of variables is more than three, we take each third-party variable as conditioning node and count the average pTE in the final results.

Q7. How to directly reproduce the numerical experiments in this research?

A7. We provided the references and access to all the data sets that we used in the research, and we also provide the codes for our PCM framework (publicly available at https://github.com/Partial-Cross-Mapping). Thus, using all these resources, one can reproduce all the results obtained in this work.

Supplementary Note 6. Additional information for real-world systems in the main text

6.1. Gene regulatory networks

In addition to the gene regulatory network shown in Fig. 4a of the main text, we investigate four other such networks, with structures shown in Supplementary Figure 12. Supplementary Figure 13 shows the detection accu-

racies versus the threshold T using both the PCM and MCM methods for the five gene regulatory networks. The ROC curves shown in the main text together with Supplementary Figure 13 demonstrate that the PCM method is remarkably effective in ascertaining direct causal links in gene regulatory networks.

6.2. Food chain of plankton species

In this analysis, the species abundances time series are measured from 12/07/1990 to 20/10/1997 with unequal sampling interval time. We interpolate the data to make equally-spaced time series with 794 points (the time unit of one time point is per 3.35 days). Supplementary Table 5 lists the p-values associated with all possible causal directions in the food chain network of three plankton species described in the main text. For all the direct links, the p-values are extremely small, signifying high statistical significance.

6.3. Air pollution and cardiovascular disease

Air pollution is believed to be one of the major causes of cardiovascular diseases. To establish this causal relation, we investigate real data sets of air pollutants and disease occurrence in the city of Hong Kong, where the collected data constitute the daily concentrations (in $\mu\mathrm{gm}^{-3}$) of nitrogen dioxide (NO_2), sulfur dioxide (SO_2), respirable suspended particulate (Rspar), and ozone (O_3) from the air-monitoring stations in Hong Kong from 1994 to 1997. Simultaneously recorded was the daily number of cardiovascular disease admissions (Cardio) into major hospitals in Hong Kong. To avoid the effect of sudden addition of hospital beds in early 1995, we choose the time series of 1000 days from March 1995 to November 1997 (the unit of one time point is per day). The corresponding p-values for all the possible causal directions with the PCM method are listed in Supplementary Table 6. Here, the significance index verified by the multi-testing corrections is set to be $p < 10^{-9}$.

Supplementary Note 7. Additional real-world examples: From gene regulatory networks to electrical information pathways

In order to further demonstrate the broad applicability of the PCM method, we investigate more real-world examples, revealing new viewpoints to the dynamical underpinnings of the real-world systems.

7.1. Reconstructing gene regulatory networks of circadian rhythm

We validate the effectiveness of our method with the real gene expression data. We consider the gene expression time series that were measured by Affimetrix microarray (Genechip Rat Genome 230 2.0) of the laboratory rat (Rattus norvegicus) cultured cells sampled from suprachiasmatic nucleus (SCN) for studying circadian rhythm [18, 19, 20, 21]. To elucidate the gene regulatory network architecture, we select the time series consisting of 16 time points, which is measured after the drug perturbation in the 19th hour (one measured time point corresponds to per 4.5 hours, more detailed information on data collection can be found in [18]). For the mammalian circadian clocks, it has been identified that there are approximately 17 genes involved in the core regulatory network, where the transcriptional circuits are formed by regulation of *E/E'* boxes, *DBP/E4BP4* binding elements, and *RevErbA/ROR* binding elements, respectively [22, 23]. Moreover, in addition to the gene-level interactions, there are also regulatory interactions at the protein level, e.g., the transcription factor *Clock* is phosphorylated by *PFK* family genes and the cyptochrome genes, *Cry1* and *Cry2*, are phosphorylated by *MAPK* family genes [18]. Thus, we consider a gene regulatory network containing 17 core circadian genes and 20 kinase genes, as shown in Supplementary Figure 14(a). We interpolate the original time series to the series of 136 points (one point per half-hour). We thus apply the PCM method to the interpolated series to detect the directional regulatory relations among all the genes and depict the corresponding ROC curve in Supplementary Figure 14(b). As a comparison, we show the ROC curves obtained by using all the other causations detection methods in Supplementary Figure 14(b) as well.

We emphasize that the inference of the gene regulatory network based only on one single and short-term series is a challenging task. The existing inference methods can usually reach a value of AUC around 0.7 for synthetic data but only around 0.5 for real experimental data [24]. This is also confirmed by our comparison study. In

fact, for the 37-genes network, the GC-based and the TE-based methods can hardly detect any structure in the regulatory networks where the value of AUC is around 0.5, while the PCM method, whose AUC value reaches 0.65, outperforms all the other methods. In a relatively large network with very dense interactions, the current PCM method also could produce false negative detection results due to the over conditioning. Some optimal technique needs to be introduced to avoid such over conditioning situation, which becomes one of our present research works.

Additionally, we further demonstrate the effectiveness of the PCM method by investigating another sub-functional regulatory network with 18 genes, the core circadian genes and the *PFK* family [see Supplementary Figure 14(c)]. Clearly, the PCM method achieves a relatively high AUC value, approximately 0.73, while all the other methods cannot produce such a good performance in causations detection.

7.2. Revealing direct electrical information pathways in the human brain through EEG dataset

This example investigates the brain dynamics through analyzing the EEG recordings, which contain time series from 10 subjects with 19 electrodes per subject at a sampling rate of 256 Hz. Each recording lasts around 1 minute. All the data and their detailed descriptions are available at: http://clopinet.com/causality/data/nolte/ [1]. The data are filtered with a stop-band at 3 Hz to remove slow drifts. The data is divided into blocks of 4 seconds (i.e., 1024 data points) during which the recording is a continuous measurement that is cleaned of apparent artifacts. For each subject, we randomly select 10 blocks, calculate the PCM measures for every pair of electrode channels in the blocks, and show the average of the detection results in Supplementary Figure 15. Since the detection results on all the subjects show homogeneous properties, we further display an average over the detection results of all the subjects in Supplementary Figure 16(a). It shows that, instead of the remote connections between the brain regions, the signals (or information) are transferred majorly through the neighboring regions. This type of neighboring connections can be regarded as direct causations detected by our PCM framework. The detected connections also reveal the direct information pathways in the human brain. For example, the left hemisphere and the right hemisphere are connected successively by the middle channels, and a directional pathway is identified from frontal to dorsal channels in Supplementary Figure 16(a). These are consistent almost with the results presented in the literature [1, 15]. However, as shown in Supplementary Figure 16(b), using the MCM method in the same manner on the EEG data produces dense connections which are replete with indirect and remote links. This thus cannot bring any information pathways in the human brain.

Supplementary References

[1] Nolte, G. *et al.* Robustly estimating the flow direction of information in complex physical systems. *Phys. Rev. Lett.* **100**, 234101 (2008).

[2] Milo, R. *et al.* Network motifs: simple building blocks of complex networks. *Science* **298**, 824–827 (2002).

[3] Alon, U. Network motifs: theory and experimental approaches. *Nat. Rev. Genet.* **8**, 450 (2007).

[4] Noble, W. S. How does multiple testing correction work? *Nat. Biotechnol.* **27**, 1135 (2009).

[5] Shaffer, J. P. Multiple hypothesis testing. *Annu. Rev. Psychol.* **46**, 561–584 (1995).

[6] Barnett, L., Barrett, A. B. & Seth, A. K. Granger causality and transfer entropy are equivalent for gaussian variables. *Phys. Rev. Lett.* **103**, 238701 (2009).

[7] Ma, H. *et al.* Detection of time delays and directional interactions based on time series from complex dynamical systems. *Phys. Rev. E* **96**, 012221 (2017).

[8] Milojevic, A. *et al.* Short-term effects of air pollution on a range of cardiovascular events in england and wales: case-crossover analysis of the minap database, hospital admissions and mortality. *Heart* **100**, 1093–1098 (2014).

[9] Duggento, A., Stankovski, T., McClintock, P. V. & Stefanovska, A. Dynamical bayesian inference of time-evolving interactions: From a pair of coupled oscillators to networks of oscillators. *Phys. Rev. E* **86**, 061126 (2012).

[10] Stankovski, T., Duggento, A., McClintock, P. V. & Stefanovska, A. A tutorial on time-evolving dynamical bayesian inference. *Eur. Phys. J-Spec. Top.* **223**, 2685–2703 (2014).

[11] Stankovski, T., Ticcinelli, V., McClintock, P. V. & Stefanovska, A. Coupling functions in networks of oscillators. *New J. Phys.* **17**, 035002 (2015).

[12] Stankovski, T., Pereira, T., McClintock, P. V. & Stefanovska, A. Coupling functions: universal insights into dynamical interaction mechanisms. *Rev. Mod. Phys.* **89**, 045001 (2017).

[13] Park, H.-J. & Friston, K. Structural and functional brain networks: from connections to cognition. *Science* **342**, 1238411 (2013).

[14] Kantz, H. & Schreiber, T. *Nonlinear Time Series Analysis*, vol. 7 (Cambridge Univ. Press, Cambridge, 2004).

[15] Harnack, D., Laminski, E., Schünemann, M. & Pawelzik, K. R. Topological causality in dynamical systems. *Phys. Rev. Lett.* **119**, 098301 (2017).

[16] Hartigan, J. A. & Wong, M. A. Algorithm as 136: A k-means clustering algorithm. *J. R. Stat. Soc. C-Appl.* **28**, 100–108 (1979).

[17] Barnett, L. & Seth, A. K. The mvgc multivariate granger causality toolbox: a new approach to granger-causal inference. *J. Neurosci. Meth.* **223**, 50–68 (2014).

[18] Wang, Y., Zhang, X.-S. & Chen, L. A network biology study on circadian rhythm by integrating various omics data. *OMICS* **13**, 313–324 (2009).

[19] Kawaguchi, S. *et al.* Establishment of cell lines derived from the rat suprachiasmatic nucleus. *Biochem. Bioph. Res. Co.* **355**, 555–561 (2007).

[20] Morioka, R. *et al.* Phase shifts of circadian transcripts in rat suprachiasmatic nucleus. In *The Second International Symposium on Optimization and Systems Biology* (Citeseer, Pennsylvania, 2008).

[21] Ma, H., Aihara, K. & Chen, L. Detecting causality from nonlinear dynamics with short-term time series. *Sci. Rep.* **4**, 7464 (2014).

[22] Ueda, H. R. *et al.* System-level identification of transcriptional circuits underlying mammalian circadian clocks. *Nat. Genet.* **37**, 187 (2005).

[23] Ko, C. H. & Takahashi, J. S. Molecular components of the mammalian circadian clock. *Hum. Mol. Genet.* **15**, R271–R277 (2006).

[24] Wang, M. *et al.* Legumegrn: a gene regulatory network prediction server for functional and comparative studies. *PLoS One* **8**, e67434 (2013).
